# Re-irradiation with hypo-fractionated stereotactic robotic radiotherapy for salvage in adult patients with brainstem glioma

**DOI:** 10.3332/ecancer.2013.366

**Published:** 2013-10-23

**Authors:** Sridhar P Susheela, Swaroop Revannasiddaiah, Sandeep Muzumder, Govindarajan Mallarajapatna, Kumar Kallur, Ajaikumar S Basavalingaiah

**Affiliations:** HealthCare Global – Bangalore Institute of Oncology, Bangalore, Karnataka 560027, India

**Keywords:** re-irradiation, brainstem glioma, reirradiation of brainstem glioma, adult brainstem glioma, stereotactic, hypofractionated

## Abstract

**Purpose:**

Brainstem glioma (BSG) is often treated with definitive irradiation. However, subsequent progression and death occur as a rule rather than the exception, after varying periods of control. The outlook of patients with post-irradiation progression is dismal, and most of these patients are treated with supportive care alone. Despite the obvious risks with an area as critical as the brainstem, it is a possibility to encounter situations wherein the patients (*themselves or their associates*) ask for re-irradiation, with the hope of a few extra months of life. The risk of radiation-induced brainstem toxicity may be justifiable under the strict assumption that the patients stand a chance of benefiting from re-irradiation but still may not live long enough to manifest brainstem toxicity.

**Methods:**

Five adult BSG patients were treated with re-irradiation using robotic-arm stereotactic radiation therapy (SRT) between September 2009 and July 2012, primarily at the request of the concerned patient parties. Re-irradiation doses ranged from 16 to 25 Gray (Gy) delivered by robotic arm stereotactic irradiation in 2–5 fractions.

**Results:**

Four out of five patients enjoyed a prolongation of survival in the order of months (three, five, six, and 14 months), which was very significant given that all patients had severe neurological compromise and poor performance status prior to re-irradiation. One patient has survived 36 months after re-irradiation and thus has lived long enough to manifest late radiation-induced brainstem toxicity.

**Conclusion:**

Despite the obvious risks of brainstem toxicity associated with the use of re-irradiation for BSG, the use of fractionated stereotactic re-irradiation seems to offers prospects of additional periods of local control and augments duration of life.

## Conflicts of interest

None

## Introduction

While gross total resection of glioma is recognised as the most crucial factor determining progression-free survival (PFS), it must be recognised that brainstem glioma (BSG) is often unresectable by the virtue of their location. Surgical procedures are limited to biopsy, decompression, or shunting. Radiotherapy (RT) is currently the cornerstone of the management of BSG, with definitive doses being 50–60 Gray (Gy) in conventional fractionation. However, irrespective of the duration of local control (LC) with RT, progression with uniformly lethal consequences occurs as a rule rather than the exception.

The outlook of BSG with post-RT progression is dismal. Surgery for decompression is feasible only in exophytic lesions, and the empirical use of agents such as temozolamide or bevacizumab often does not yield meaningful responses. The use of re-irradiation is mostly avoided given the concerns regarding brainstem toxicity.

It must be recognised that most BSG patients respond well to initial RT and hence lead to a perception of RT’s potential efficacy against the condition. However, when disease progression occurs, it is not uncommon for these patients (meaning the patients themselves or their associates) to ask for “radiation again.”

We describe the use of re-irradiation for BSG in five patients suffering from progression of disease after initial RT. All patients were refused the option of re-irradiation in various other centres before they presented to us asking for re-irradiation. Although each of these patients were made aware of the risks of brainstem toxicity, they were strong minded in their demand for re-irradiation, which they justified stating the otherwise quickly fatal outcome if supportive care alone were to be used. The potential prospects of immediate neurological symptom alleviation, as well as the possible benefit in prolonging survival by a few months, were ostensibly significant enough to be worth the risk of neurotoxicity.

Furthermore, given our assumption that re-irradiation would only prolong survival by a few months, we expected no risk in the development of severe brainstem toxicity. We thus utilised a robotic-arm stereotactic radiosurgery system (CyberKnife^TM^, Accuray, Sunnyvale, California, USA) to deliver re-irradiation via fractionated stereotactic radiotherapy (FSRT).

## Patient and treatment characteristics

The characteristics of the five patients who had been previously diagnosed with BSG are presented in Table 1. The patients were adults aged between 24 and 63 years. All BSG were of the intrinsic type, with initial histology including both low-grade and high-grade gliomas. Three patients who had lower histological grades during their initial diagnosis had higher-grade gliomas during re-biopsy at progression.

Four patients had received previous RT with conventional fractionation via parallel opposed beams (50 Gy in 25 fractions in three patients, 50.4 Gy in 28 fractions in one patient), while one patient had been previously treated with FSRT to a dose of 25 Gy in five fractions. As expected in view of the natural history of the disease, after varying periods of disease control, all patients had experienced subsequent progression and neurological deterioration. The performance status of patients at presentation for re-irradiation was documented per the Eastern Cooperative Oncology Group (ECOG) performance status scale [1].

All patients were informed about the potential risks of brainstem toxicity prior to the initiation of re-irradiation. Re-irradiation in all patients was done with the CyberKnife^TM ^robotic-arm stereotactic radiosurgery system. The time gap from previous irradiation to re-irradiation ranged from 12 to 26 months.

Target volumes ranged from 30 to 55 cm^3^. The chosen prescription isodose ranged from 72% to 85%. The prescribed doses were 20 Gy delivered in five consecutive daily fractions in three patients (prescription isodose lines at 72%, 78%, 79%), while it was 25 Gy in five fractions to one patient (prescription isodose line at 82%) and 16 Gy in two fractions to another (prescription isodose line at 85%).

Patient 1, who had been diagnosed with fibrillary astrocytoma of the pons and midbrain had previously undergone conventional fractionated RT to a dose of 50 Gy in 25 fractions, after which he was diagnosed with progression to glioblastoma multiforme (GBM), and initiation of chemotherapy with temozolamide did not yield symptomatic or radiological benefit. The patient then underwent re-irradiation (23 months after prior irradiation), in spite of which the patient did not survive beyond three months. The patient had transiently improved but ultimately succumbed to multiorgan failure, complicated by pre-existing diabetes mellitus.

Patient 2 had received prior treatment with stereotactic irradiation (25 Gy/in five fractions) for a pontine GBM. On relapse, re-irradiation was done stereotactically (13 months after prior irradiation) to a dose of 25 Gy in five fractions. Although there was a transient improvement in performance status, the patient succumbed to progressive disease after three months.

Patient 3, who was initially treated with conventionally fractionated RT to a dose of 50.4 Gy in 28 fractions subsequently suffered progression of disease and was re-irradiated with 25 Gy in five fractions (12 months after initial RT). The patient is alive 30 months after re-irradiation, although it must be noted that this particular patient has suffered restricted activity during the past ten months, including generalised weakness and lack of coordination, which could very well be attributed to radiation toxicity.

Patient 4 had received prior treatment with conventional RT to a dose of 50 Gy in 25 fractions and on relapse was re-irradiated (22 months after prior irradiation) with 16 Gy in two fractions to the progressive brainstem disease. Since the patient had also developed multicentric GBM in the cerebrum, he was also treated with whole-brain RT (45 Gy in 25 fractions) with a stereotactic RT boost of 16 Gy in two fractions to a separate frontal lobe lesion, and the patient was alive for the next 14 months (while also taking temozolamide).

Patient 5 was treated earlier for anaplastic astrocytoma involving the pons and the medulla, with conventional RT to a dose of 50 Gy in 25 fractions. Re-irradiation was done with stereotactic irradiation to a dose of 20 Gy in five fractions after a gap of 26 months from prior irradiation. There was a marked improvement in the performance status, and the patient remains alive about a year after re-irradiation. There have been no major signs of radiation-induced brainstem toxicity as of yet.

After re-irradiation all but one patient were noticed to manifest an improvement in ECOG performance status scores on the first follow-up visit one month after re-irradiation (Table 2). As far as the patients’ perceptions of the improvements were concerned, four out of five patients felt an improvement in their general condition.

## Discussion

The treatment of BSG, especially of the diffuse and the intrinsic varieties, involves the use of RT as the mainstay [2–4]. However, conventionally delivered doses of up to 60 Gy cannot be expected to achieve cure, or long-term survival in these patients [5–7]. Subsequent progression occurs, leading to neurological deterioration and ultimately death.

The decision to re-irradiate the brainstem area, which in itself is regarded as an intracranial dose-limiting structure, cannot be made routinely. However, given that death is otherwise inevitable, the risk of brainstem toxicity may seem justifiable to many patients and their associates.

Given that many patients who develop progressive disease after initial RT also manifest an increase in tumour grade, we expected that the use of re-irradiation may confer short-term benefits, which may not be lengthy enough to allow the manifestation of brainstem toxicity.

When the use of re-irradiation is decided upon, the use of stereotactic irradiation has the advantages of high conformity and short treatment times. Given that the target volumes were small (Table 2), the purpose of re-irradiation was well fulfilled by our use of robotic-arm stereotactic radiosurgery.

The overall outcomes in the five described patients seem to be encouraging. All but one patient enjoyed survival periods in the order of months, which is a very significant result given that all the patients had severe neurological compromise and poor performance status prior to the initiation of re-irradiation. However, on the other hand, one of the patients who survived 26 months after re-irradiation has during the past six months been suffering from generalised weakness and loss of coordination. This particular patient subsequently suffered progression, and has now been transitioned to supportive care alone.

Given that this analysis involves only five patients, it cannot be expected to provide ground for us to field definitive conclusions. We suggest a pooled analysis of similar patients treated in various centres around the world. Also, until a more acceptable treatment is available for progressive BSG, we believe that re-irradiation with fractionated stereotactic RT holds the potential to offer modest survival benefits. Thus, re-irradiation should not be denied for patients who opt for the same after understanding the risks involved. Although this study was confined to adult patients, the published literature already supports the use of re-irradiation among paediatric recurrent BSGs [8].

## Conclusion

The risk of brainstem toxicity is ever present with the use of re-irradiation for BSG. But, given that deterioration and death are otherwise inevitable, the use of re-irradiation with FSRT may offer a new lease of LC and PFS that could span for months. The act of choosing between a potential benefit against the consequence of toxicity is best done in collaboration with the patient and their associates. The ideal dose-fractionation schedules and the true potential of this difficult approach may be learnt better from a pooled analysis of the outcomes with similar patients from multiple centres.

## Figures and Tables

**Table 1.** Patient details.

			Diagnosis			Histology on relapse ^d^	ECOG before ReRT ^e^	Previous RT	
Pt. No.	Age ^a^	Sex	Type	Site ^b^	Initial Hist ^c^			Period	Dose
1	33	M	Intrinsic	*P,Mi *	*FA *	GBM	4	Jan 2008	50 Gy/25 Fx
2	63	M	Intrinsic	*P *	*GBM *	GBM	2	Mar 2010	25 Gy/5 Fx SRT
3	24	M	Intrinsic	*P,Mi *	*PA *	AA	4	Apr 2009	50.4 Gy/28 Fx
4	24	M	Intrinsic	*P,Me *	*AA *	AA	3	Nov 2008	50 Gy/25 Fx
5	34	F	Intrinsic	*P *	*FA *	GBM	2	May 2010	50 Gy/25 Fx

RT, radiotherapy; ECOG, Eastern Cooperative Oncology Group performance status; AA, anaplastic astrocytoma;

PA, pilocytic astrocytoma; FA, fibrillary astrocytoma; GBM, glioblastoma multiforme.

a Age at initial RT.

b Site at initial diagnosis (all intrinsic type); *P*: Pons; *Me*: Medulla; *Mi*: Midbrain.

c Histology at initial diagnosis.

d Repeat histology at the time of re-irradiation.

e ECOG performance status scores at the time of presentation for re-irradiation.

**Table 2.** Stereotactic dose prescriptions and outcomes.

Pt. No.	Month of re-irradiation	Time since previous RT (months)	TV (mm^3^)^a^	Dose^b^	PI (%)	ECOG after one month	Subjective Improvement^c^	Survival in months after Re-RT
1	Sep 2009	21	33015	20 Gy (5 Fx)	78	2	Yes	Died at six months
2	Mar 2010	13	30056	25 Gy (5 Fx)	82	3	Yes	Died at three months^e^
3	Mar 2010	12	39180	20 Gy (5 Fx)	72	2	No	Alive after 36 months^f^
4	Sep 2010^d^	22	46459	16 Gy (2 Fx)^+^	85	1	Yes	Died at 14 months
5	Jul 2012	26	54883	20 Gy (5 Fx)	79	1	Yes	Alive at 12 months

RT, radiotherapy; PI, prescription isodose line; ECOG, Eastern Cooperative Oncology Group performance status.

a Target volume in cubic millimetres.

b Doses in Gy in continuous daily fractions.

c No formal questionnaires were used to assess ‘subjective improvement’. Patient/associate satisfaction at first month post re-irradiation is presented.

d Patient no. 4 required further whole-brain RT in view of multiple lesions consistent with glioblastoma multiforme.

e Patient died at three months due to complications of diabetes mellitus.

f Patient although alive at 30 months, however, has now suffered another relapse in the brainstem and cerebellum.
